# AKR1C1/2 inhibition by MPA sensitizes platinum resistant ovarian cancer towards carboplatin

**DOI:** 10.1038/s41598-022-05785-9

**Published:** 2022-02-03

**Authors:** Susann Badmann, Doris Mayr, Elisa Schmoeckel, Anna Hester, Christina Buschmann, Susanne Beyer, Thomas Kolben, Fabian Kraus, Anca Chelariu-Raicu, Alexander Burges, Sven Mahner, Udo Jeschke, Fabian Trillsch, Bastian Czogalla

**Affiliations:** 1 Department of Obstetrics and Gynecology, University Hospital, LMU Munich, Marchioninistr 15, 81377 Munich, Germany; 2 Institute of Pathology, Faculty of Medicine, LMU Munich, Munich, Germany; 3 Department of Obstetrics and Gynecology, University Hospital Augsburg, Augsburg, Germany

**Keywords:** Gynaecological cancer, Ovarian cancer, Cancer therapeutic resistance

## Abstract

In recurrent epithelial ovarian cancer (EOC) most patients develop platinum-resistance. On molecular level the NRF2 pathway, a cellular defense mechanism against reactive oxygen species, is induced. In this study, we investigate AKR1C1/2, target of NRF2, in a well-established EOC collective by immunohistochemistry and in a panel of ovarian cancer cell lines including platinum-resistant clones. The therapeutic effect of carboplatin and MPA as monotherapy or in combination was assessed by functional assays, using OV90 and OV90cp cells. Molecular mechanisms of action of MPA were investigated by NRF2 silencing and AKR activity measurements. Immunohistochemical analysis revealed that AKR1C1/2 is a key player in the development of chemoresistance and an independent indicator for short PFS (23.5 vs. 49.6 months, p = 0.013). Inhibition of AKR1C1/2 by MPA led to a concentration- and time-dependent decline of OV90 viability and to an increased response to CP in vitro. By NRF2 silencing, however, the effects of MPA treatment were reduced. Concludingly, our data suggest that a combination therapy of carboplatin and MPA might be a promising therapeutic approach to increase response rates of EOC patients, which should be explored in clinical context.

## Introduction

Ovarian cancer is the most lethal gynecologic malignancy with a five-year survival rate of less than 45% over all Federation of Gynecology and Obstetrics (FIGO) stages^1,2^. It is mostly diagnosed in advanced disease (FIGO III/IV) because of unspecific symptoms and insufficient screening methods^3^. The standard of care for advanced EOC consists of radical cytoreductive surgery followed by adjuvant platinum-based chemotherapy and maintenance targeted therapy such as anti-angiogenic antibody, bevacizumab, or poly-ADP-ribose-polymerase inhibitors^4^. Even though initial response rates are between 60 and 80%, the majority of patients will develop resistance, leading to subsequent recurrence or progression of disease. Therefore, given the rising platinum resistance over the clinical course^5^, translational research approaches are needed to elucidate the molecular mechanisms of the development of chemoresistance and to find effective therapeutic options for patients with recurrent disease.

Chemotherapeutic agents generate oxidative stress, which contributes significantly to the destruction of tumor cells^6,7^. There is increasing evidence that the NRF2-mediated antioxidant response pathway, which is considered to be the primary cellular defense mechanism against reactive oxygen species (ROS), is activated during the development of chemoresistance^8,9^. While NRF2 is ubiquitously expressed at low levels in all human tissues, elevated levels are found in different cancer entities including ovarian cancer^10–16^. Our group recently demonstrated that NRF2 in its inactive form is significantly correlated with improved overall survival of ovarian cancer patients^17^. Given our previous data, we hypothesized that the downstream gene of NRF2 pathway, *AKR1C1*, might protect cancer cells from cytotoxic effects of chemotherapy through its inhibitory effect on progesterone metabolism.

Amongst others, NRF2 activates the reductases AKR1C1 and 2 via an antioxidant response element in the promotor region, which subsequently detoxify ROS^7,18^. Both enzymes share high sequence identity and play important roles in human steroid hormone and xenobiotic metabolism with varying expression patterns and substrate preferences^19,20^. AKR1C1 is mainly expressed in the human ovary, which preferentially catalyzes the inactivation of progesterone^21^. An upregulation of AKR1C1/2 enzymes was seen especially in hormone dependent cancer types, including prostate or endometrial carcinoma. An upregulation of AKR1C1/2 in benign conditions such as ovarian endometriosis was reported as well^22–24^.

For EOC a protective effect of combined oral contraceptives has been described^25^. In contrast, postmenopausal hormone replacement therapy, especially the application of estrogen-only agents, is associated with increased risk of EOC^26^. Antiproliferative, anti-inflammatory, and anticarcinogenic properties are attributed to progesterone with limiting effects on tumor growth and metastasis^27–29^. Furthermore, progesterone is reported to facilitate the toxicity of cisplatin in EOC cells and xenografts^30^.

In this study we focus on the NRF2-AKR1C1/2 pathway to further characterize the molecular basis of chemoresistance and propose as therapy option in recurrent ovarian cancer carboplatin in combination with the synthetic progesterone MPA.

## Material and methods

### Patient cohort

Pathologically confirmed EOC tissue samples of 156 patients that underwent radical cytoreductive surgery between 1990 and 2002 at the Department of Gynecology and Obstetrics, Ludwig-Maximilians-University, Munich, Germany were analyzed in this study. The study was approved by the Ethics Committee of the Faculty of Medicine, Ludwig-Maximilians-University, Munich, Germany (approval number 227-09, 18-392, and 19-972). The tissue samples derive from the archives of the Department of Gynecology and Obstetrics, Ludwig-Maximilians-University, Munich, Germany and were initially used for histopathological diagnostics. Before the current study was performed, all diagnostic procedures have been completed and the patients’ data were fully anonymized. Due to these circumstances the Ethics Committee of the Faculty of Medicine, Ludwig-Maximilians-University, Munich, Germany declared that no written informed consent of the participants or permission to publish is needed. The standards of the Declaration of Helsinki 1975 have been respected.

Except for patients in stage FIGO IA with low-grade histology, all patients received adjuvant platinum-based chemotherapy. The EOC specimens have been observed by specialized gynecologic pathologists at the Department of Pathology, Ludwig-Maximilians-University, Munich, Germany, who determined grading and histological subtype. Grading of mucinous subtype was performed analogous to endometrioid subtype. Staging was performed according to the FIGO (2014) and TNM classification. Clinical data derived from our patients’ charts, follow up data were received from the Munich Cancer Registry. The clinicopathologic characteristics of the patients are listed in Table 1. Information to FIGO stage and grading are missing in 5 and 9 cases respectively.

**Table 1:** Clinicopathologic characteristics of the analyzed EOC patients.

Clinicopathologic parameters	n	Percentage (%)
**Histology**		
Serous	110	70.5
Clear cell	12	7.7
Endometrioid	21	13.5
Mucinous	13	8.3
**Primary tumor expansion**		
TX	1	0.6
T1	40	25.6
T2	18	11.5
T3	97	62.3
**Nodal status**		
pNX	61	39.1
pN0	43	27.6
pN1	52	33.3
**Distant metastasis**		
pMX	147	94.2
pM0	3	1.9
pM1	6	3.8
**Grading serous**		
Low	24	21.8
High	80	72.7
**Grading endometrioid**		
G1	6	28.6
G2	5	23.8
G3	8	38.1
**Grading mucinous**		
G1	6	46.2
G2	6	46.2
G3	0	0
**Grading clear cell**		
G3	12	100.0
**FIGO**		
I	35	22.4
II	10	6.4
III	103	66.0
IV	3	1.9
**Age**		
≤ 60 years	83	53.2
> 60 years	73	46.8

### Immunohistochemistry

Tissue microarrays with three representative biopsies (0.6 mm in diameter) of each formalin-fixated and paraffin-embedded tumor sample were stained for immunohistochemistry (IHC) analysis according to a protocol previously published by our lab^31^. After dewaxing, rehydration and thermal demasking of the tissue slides, AKR1C1/2 expression was visualized using a polyclonal rabbit IgG (dilution: 1:100, 16 h at 4 °C) (abcam, Cambridge, UK) as primary antibody and the ZytoChem-Plus HRP Polymer-Kit (Zytomed Systems GmbH, Berlin, Germany) with 3,3’-Diaminobenzidine (Dako, Carpinteria, CA, USA) as chromogenic substrate. Mayer’s hemalum (Waldeck GmbH & Co. KG, Münster, Germany) was used for counterstaining. Tissue from human kidney served as system control to assess the specificity of the immunoreactions (Supplementary Fig. S1). Staining evaluation has been performed by two independent observers in a double-blind process using a photomicroscope (Leitz, Wetzlar, Germany). The semiquantitative immunoreactive score (IRS) was determined by multiplying the intensity of AKR1C1/2 staining (0: no, 1: weak, 2: moderate, and 3: strong staining) and the percentage of positive cells (0: no staining, 1: $\le$ 10%, 2: 11–50%, 3: 51–80% and 4: $\ge$ 81% positive)^32^. Mean IRS of the three representative biopsies from one patient were used for further analyses. The descriptive statistics of the expression analysis is shown in the Supplementary Table S1.

### Statistical analysis

Data processing, statistical analysis, and graphics have been performed using SPSS 26.0 (IBM Corporation, Armonk, NY, USA) and MATLAB R2020b (The MathWorks, Natick, MA, USA). Kruskal Wallis H-test was used to compare the distribution of more than two independent samples^33^, t-test was applied to compare the means of independent groups. Previously, other pathological markers including NRF2 and the hormone receptors were investigated in the same patient cohort, which enables correlation analysis^16,17^. Bivariate correlations between clinical and pathological data have been calculated with Spearman’s analysis^34^. Expression-dependent differences in survival times were detected by log-rank testing and visualized in Kaplan-Maier curves^35^. Appropriate cut-off points considering the distribution pattern of IRS in the collective have been determined by ROC analyses and maximizing the Youden index^36,37^. Cox regression models were used for multivariate analyses^38^. P-values ≤ 0.05 were considered as statistically significant.

### Cell lines and tissue culture

The endometrioid cell line A2780 and the serous cell lines CaOV3, COV318, OV90, OVCAR3, and SKOV3 were purchased from ATCC (Manassas, VA, USA). OV90 and its platinum-resistant clone OV90cp were cultured in Dulbecco’s Modified Eagle’s Medium F-12 (Gibco, Paisley, UK) supplemented with 10% fetal bovine serum (Gibco, Paisley, UK) in a humified incubator at 37 °C under 5% CO_2_. The other cell lines were maintained in RPMI 1640 GlutaMAX medium (Gibco, Paisley, UK) under the same conditions. Platinum-resistant clones were induced by continuous increase of carboplatin (CP) (0.1, 0.2, 0.33, 0.5 µM) concentration up to 1 µM over 5 weeks. The chemoresistance was confirmed by determination of the half maximal inhibitory concentration (IC50). To maintain the resistance the medium was supplemented with 1 µM CP. No antibiotics or antimycotics were used.

### Reagents

Medroxyprogesterone acetate (MPA) (Sigma-Aldrich Co., St. Louis, MO, USA) is a clinically used progestin. Besides its effects on hormone receptors (progesterone receptor agonism and estrogen receptor antagonism), it is considered as pan-AKR1C inhibitor^21^. For in vitro experiments we used CP purchased from medac GmbH (Wedel, Germany).

### Western Blotting

Western blot analysis was performed as previously described^39^. In short, cells were lysed on ice with RIPA buffer (Sigma-Aldrich Co., St. Louis, MO, USA). Bradford protein assay was performed to determine the protein concentration of the lysates. Equal amounts of protein were separated according to their molecular weight by SDS gel electrophoresis and transferred onto a polyvinylidene fluoride membrane. After blocking to prevent non-specific binding, the primary antibody against NRF2, AKR1C1/2, and β-actin (Supplementary Table S2) was incubated overnight at 4 °C. β-actin served as control. Western blot detection was performed with VECTASTAIN ABC-AmP Reagent (Vector Laboratories, Burlingame, CA, USA). Pictures were captured with Bio-Rad Universal Hood II (Bio-Rad Laboratories Inc., Hercules, CA, USA) and the corresponding Software Quantity One enabled a quantitative analysis of the blots. Each Western blot experiment was conducted 3 times. The mean optical density normalized to the background of each blot is shown in the graphs.

### qPCR

According to the instructions of the manufacturer RNA was isolated with the RNeasy Mini Kit (QIAGEN, Venlo, Netherlands). 1 µg was converted into first-strand cDNA using the cDNA Synthesis Kit (Biozym Scientific GmbH, Hessisch Oldendorf, Germany). To quantify mRNA expression of *NFE2L2* and *AKR1C1/2*, qPCR was performed using the FastStart Essential DNA Probes Master (Roche, Basel, Switzerland) and gene-specific primers (Supplementary Table S3) (Eurofins Genomics, Ebersberg, Germany). *ACTB* and *GAPDH* served as housekeeping genes. Relative expressions were calculated using the 2^-ΔΔCt^ method. Statistical analysis was performed based on data from 3 biological and 3 technical replicates each.

### siRNA knockdown

To silence *NFE2L2* OV90/OV90cp cells were transfected with Silencer Select siRNAs (si91 and si93; Ambion, Carlsbad, CA, USA) using Lipofectamine RNAiMAX Reagent (Invitrogen, Carlsbad, CA, USA). Efficiency of the siRNA knockdown was validated by qPCR and western blotting 24 h after transfection. A scrambled siRNA served as negative control (siCo).

### Cell viability and proliferation

OV90/OV90cp and A2780/A2780cp cells (10^4^ cells/well) were seeded into microplates. Once adherent, the cells were treated with different concentrations of CP for IC50 determination, MPA (0, 1, 5, 10 µM), or a combination of both agents. Cell viability was determined by a spectrophotometric method after 24, 48 or 72 h treatment. Metabolically active cells reduce yellow 3–(4,5-dimethylthiazol-2-yl)-2,5-diphenyltetrazolium bromide (MTT) (Sigma-Aldrich Co., St. Louis, MO, USA) to purple formazan crystals, which is measured at 595 nm. Cell proliferation was quantified indirectly by a colorimetric immunoassay (Cell proliferation ELISA, Roche, Basel, Switzerland) based on incorporation of the thymidine analog 5-bromo-2′-deoxyuridine (BrdU) during DNA synthesis after 48 h. The assays were carried out according to the manufacturers’ instructions.

### Apoptosis

Cell death was quantified by photometric determination of cytoplasmic histone-associated-DNA-fragments (late apoptosis marker) using Cell Death Detection ELISA^PLUS^ (Roche, Basel, Switzerland), a quantitative sandwich-enzyme-immunoassay. OV90/OV90cp cells (10^4^ cells/well) were grown in microplates overnight. Apoptosis was induced by MPA, CP or combination at different concentrations for 72 h. The assay was performed according to the manufacturer’s protocol. The apoptotic index was calculated as an enrichment factor of nucleosomes in the cytoplasm by dividing the absorbance of the sample cells by the absorbance of the control (cells without treatment).

### AKR activity

Enzyme activity of aldo-ketoreductases was measured in cell lysates of OV90/OV90cp and A2780/A2780cp (10^6^ cells) by a colorimetric AKR Activity Assay Kit (abcam, Cambridge, UK). The generation of NADPH, which is proportional to the turnover of an AKR specific substrate (AKR activity) in the sample, was detected as OD at 450 nm.

## Results

### AKR1C1/2 expression is elevated in serous and endometrioid EOC and correlates positively with NRF2 and hormone receptor expression

**Figure 1:**
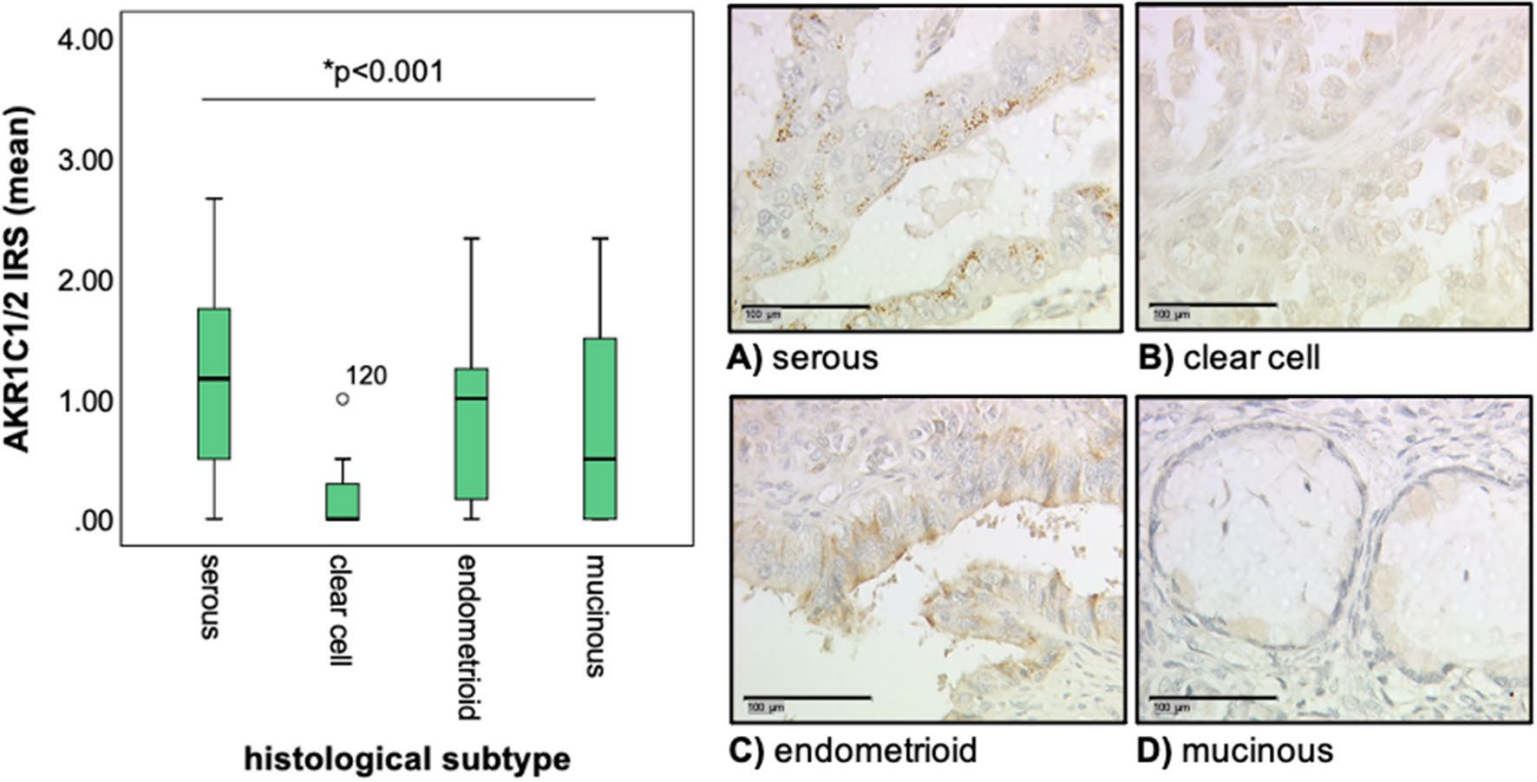
Detection of AKR1C1/2 by IHC. Representative microphotographs (25 × magnification, scale bar = 100 µm) of the AKR1C1/2 staining of EOC tissue samples with serous (**A**), clear cell (**B**), endometrioid (**C**) and mucinous (**D**) histology. Comparison of mean IRS between the histological subtypes showed significantly higher expression levels of AKR1C1/2 in serous and endometrioid EOC samples compared to clear cell and mucinous histology (p < 0.001).

AKR1C1/2 expression was analyzed in a well-established EOC collective by IHC. The tissue of 115 patients (82.1% of all evaluable cases) stained positively for AKR1C1/2. Immunoquantitative comparison of AKR1C1/2 expression between the histological subtypes revealed a significantly higher expression in serous and endometrioid EOC (Fig. 1). While AKR1C1/2 is expressed in low grade as well as high grade serous ovarian cancer at the same level, endometrioid G3 carcinomas show lower expression levels than G1. Since NRF2 is the regulating transcription factor of *AKR1C1/2*, we further explored the expression of AKR1C1/2. Our results showed a strong positive correlation between NRF2 and AKR1C1/2 expression (cc = 0.338, p < 0.001). In addition, both NRF2 and AKR1C1/2 expression strongly correlated with the expression of hormone receptors, including ERalpha, PGRA, and PGRB. These data suggest an association between hormone receptor- and NRF2-AKR1C1/2-mediated signaling (Table 2). Graphic representations of the correlations are shown in the Supplementary Figure S2.

**Table 2:** Correlation analysis between NRF2, AKR1C1/2 and the hormone receptors.

Staining	NRF2	AKR1C1/2	ERalpha	ERbeta	PGRA	PGRB
**NRF2**						
cc	1	0.338**	0.182*	0.135	0.194*	0.214**
p	–	< 0.001	0.026	0.103	0.019	0.009
n	118	112	150	147	146	148
**AKR1C1/2**						
cc	0.338**	1	0.494**	0.117	0.334**	0.203*
p	< 0.001	–	< 0.001	0.174	< 0.001	0.017
n	112	112	112	137	136	138
**ERalpha**						
cc	0.182*	0.494**	1	0.058	0.236**	0.237**
p	0.026	< 0.001	–	0.475	0.003	0.003
n	150	112	156	153	152	154
**ERbeta**						
cc	0.135	0.117	0.058	1	0.234**	0.133
p	0.103	0.174	0.475	–	0.004	0.104
n	147	137	153	153	150	152
**PGRA**						
cc	0.194*	0.334**	0.236**	0.234**	1	0.622**
p	0.019	< 0.001	0.003	0.004	–	< 0.001
n	146	136	152	150	152	152
**PGRB**						
cc	0.214**	0.203*	0.237**	0.133	0.622**	1
p	0.009	0.017	0.003	0.104	< 0.001	–
n	148	138	154	152	152	154

IRS of NRF2 and AKR1C1/2 (mean) were correlated to each other and to the IRS of hormone receptors using Spearman’s correlation analysis. Significant correlations are indicated by asterisks (*p < 0.05; **p < 0.01).

*Cc* correlation coefficient, *p* two-tailed significance, *n* number of patients.

### Patients with AKR1C1/2 expression show a significantly impaired PFS

**Figure 2:**
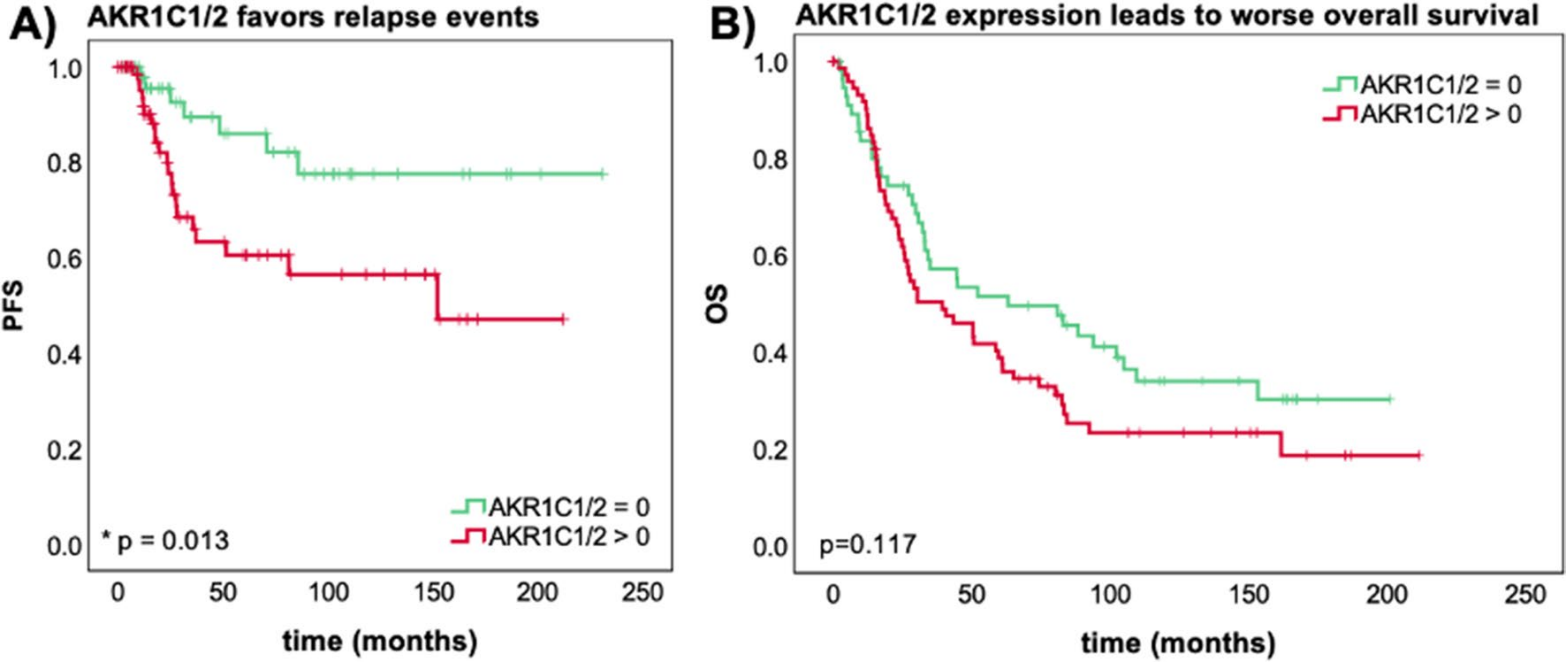
AKR1C1/2 expression has a negative effect on survival. The Kaplan–Meier estimates show PFS (**A**) and OS (**B**) depending on AKR1C1/2 expression (log-rank test). (**A**) Patients with AKR1C1/2 expression (AKR1C1/2 IRS > 0; n = 74) in their tumor tissue show a significantly shorter PFS with a median of 23.5 months compared to patients without relevant AKR1C1/2 expression (AKR1C1/2 IRS = 0; n = 50; median PFS = 49.6 months). (**B**) A similar trend is observed for OS (AKR1C1/2 IRS > 0; n = 75; median OS = 39.6 months vs. AKR1C1/2 IRS = 0; n = 56; median OS = 63.4 months). Censoring events have been marked in the graphs (+).

The prognostic impact of AKR1C1/2 expression on overall survival (OS) and progression free survival (PFS) was investigated by univariate and multivariate analyses. The median age of patients at the time of tumor debulking surgery was 58.7 years (interquartile range (IQR) = 16.4) with a total range of 20.7 to 88.0 years. Median follow-up PFS was 27.8 months (IQR = 78.0), median follow-up OS was 33.8 months (IQR = 78.3). Consistent with our hypothesis that AKR1C1/2 favors relapse events, a significantly shorter PFS was noted for patients expressing AKR1C1/2 (IRS > 0) in their tumor tissue (median PFS 23.5 vs. 49.6 months, p = 0.013, n = 124) (Fig. 2A). A similar trend was observed for OS, although the difference did not reach statistical significance (median OS 39.6 vs. 63.4 months, p = 0.117, n = 131) (Fig. 2B).

Multivariate analysis proved independency of AKR1C1/2 expression (IRS > 0) as indicator for shortened PFS (p = 0.003). However, for OS AKR1C1/2 expression did not reach statistical significance as prognostic factor in the studied patient collective (p = 0.064). Other independent prognostic factors in the present patient cohort are subtype and FIGO for PFS and age and FIGO for OS (Table 3).

**Table 3:** AKR1C1/2 expression is an independent negative prognostic factor for PFS. A cox regression model was established for multivariate analysis.

Covariate		p	Hazard Ratio (95% CI)
Subtype (serous vs. others)	OS	0.624	1.167 (0.630–2.162)
	PFS	0.047*	0.375 (0.143–0.985)
Age ($\le$ 60 vs. > 60)	OS	0.009**	1.809 (1.160–2.821)
	PFS	0.643	1.208 (0.544–2.682)
FIGO (I–II vs. III–IV)	OS	< 0.001**	3.628 (1.881–6.997)
	PFS	0.001**	9.422 (2.537–34.985)
AKR1C1/2 (0 vs. $\ge$ 1)	OS	0.064	1.560 (0.974–2.499)
	PFS	0.003**	4.384 (1.666–11.533)

Significant independent factors are indicated by asterisks (*p < 0.05; **p < 0.01).

*CI* confidence interval.

### AKR1C1/2 expression is elevated in ovarian cancer cells and in their platinum resistant clones

**Figure 3:**
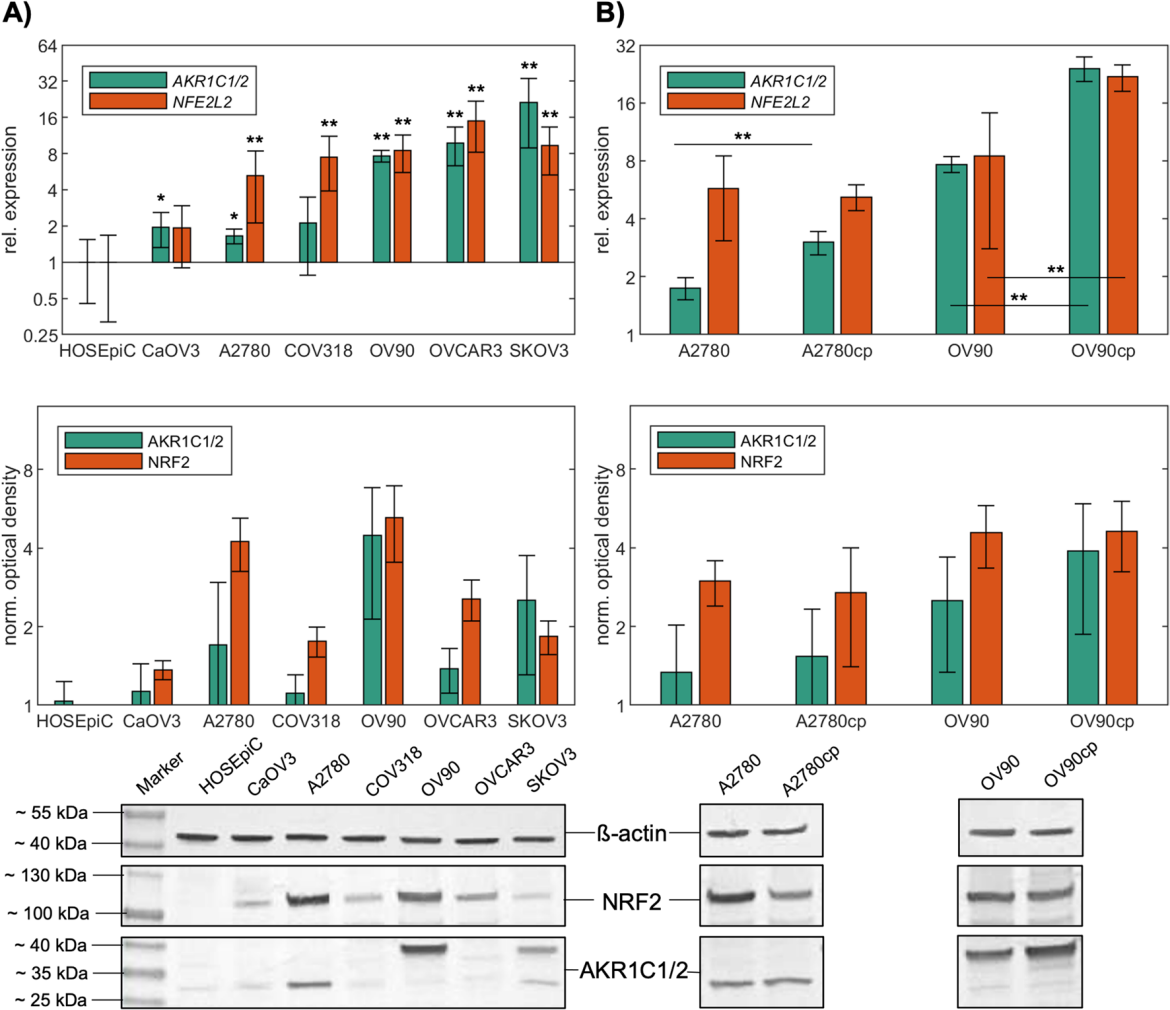
AKR1C1/2 expression is elevated in ovarian cancer cells and induced in their platinum resistant clones. (**A**) Basal mRNA (qPCR; scaled to HOSEpiC; top) and protein (western blotting; normalized to the background; bottom) expression of *NFE2L2*/NRF2 and AKR1C1/2 of ovarian cancer cell lines compared to the benign ovarian epithelial cell line HOSEpiC. (**B**) mRNA (qPCR; scaled to HOSEpiC; top) and protein (western blotting; normalized to the background; bottom) expression of *NFE2L2*/NRF2 and AKR1C1/2 of the endometrioid EOC cell line A2780 and the serous EOC cell line OV90 and their resistant clones. Full length blots are included in the Supplementary Fig. S4. *p < 0.05; **p < 0.01.

We first determined the basal expression of NRF2 and AKR1C1/2 in a panel of well-established ovarian cancer cell lines. All ovarian cancer cell lines tested had substantially higher NRF2 and AKR1C1/2 mRNA and protein levels than the benign ovarian epithelial cell line HOSEpiC (Fig. 3A). Compared to other ovarian cancer cell lines OV90 and SKOV3 have been described as relatively resistant^40^. Interestingly, OV90 and SKOV3 show the full length AKR1C1/2 protein with a molecular weight between 35 and 40 kDa, while other cell lines express smaller AKR1C1/2 splice variants between 25 and 35 kDa (Fig. 3A, bottom)^41^.

To further investigate the role of NRF2-AKR1C1/2 signaling in the development of chemoresistance, platinum-resistant clones of OV90 and A2780 were induced by continuous treatment with increased CP concentration. Subsequently, the chemoresistance was determined using the IC50 (Supplementary Figure S3) and the aldoketoreductase (AKR) enzyme activity (Table 4). IC50 of the induced clones OV90cp and A2780cp was 1.6-fold respectively 2.3-fold higher than the IC50 of the original cell lines. When OV90 is compared to A2780, OV90 was 6.4-fold more resistant towards CP and showed an 8.3 times higher AKR enzyme activity. Similar ratios are obtained for their resistant counterparts. In addition, a higher mRNA and protein expression of NRF2 and AKR1C1/2 was observed in OV90cp compared with A2780cp (Fig. 3B). When comparing the original cell lines with the resistant clones, it is noticed that especially AKR1C1/2 was induced significantly on RNA and protein level. However, for NRF2 no clear upregulation was shown. Taken together, these results indicate that the expression of AKR1C1/2 was likely induced in both cell lines during the emergence of platinum-resistance. Given our results thus far, we selected OV90 and its platinum resistant clone, OV90cp, for further functional experiments. Furthermore, since high grade serous ovarian cancer is the most common and aggressive histological subtype OV90/OV90cp was considered to be a suitable model to investigate chemoresistance, due to its serous origin as well.

**Table 4:** Characteristics of A2780 and OV90 and their platinum resistant clones.

Cell line	IC50 [µM]	Resistance relative to A2780	AKR activity [mU/ml]	AKR activity relative to A2780
A2780	282	1	0.30	1
A2780cp	645	2.3	0.31	1.013
OV90	1791	6.4	2.52	8.289
OV90cp	2826	10	2.54	8.342

### MPA directly regulates AKR activity and impacts OV90 viability

**Figure 4:**
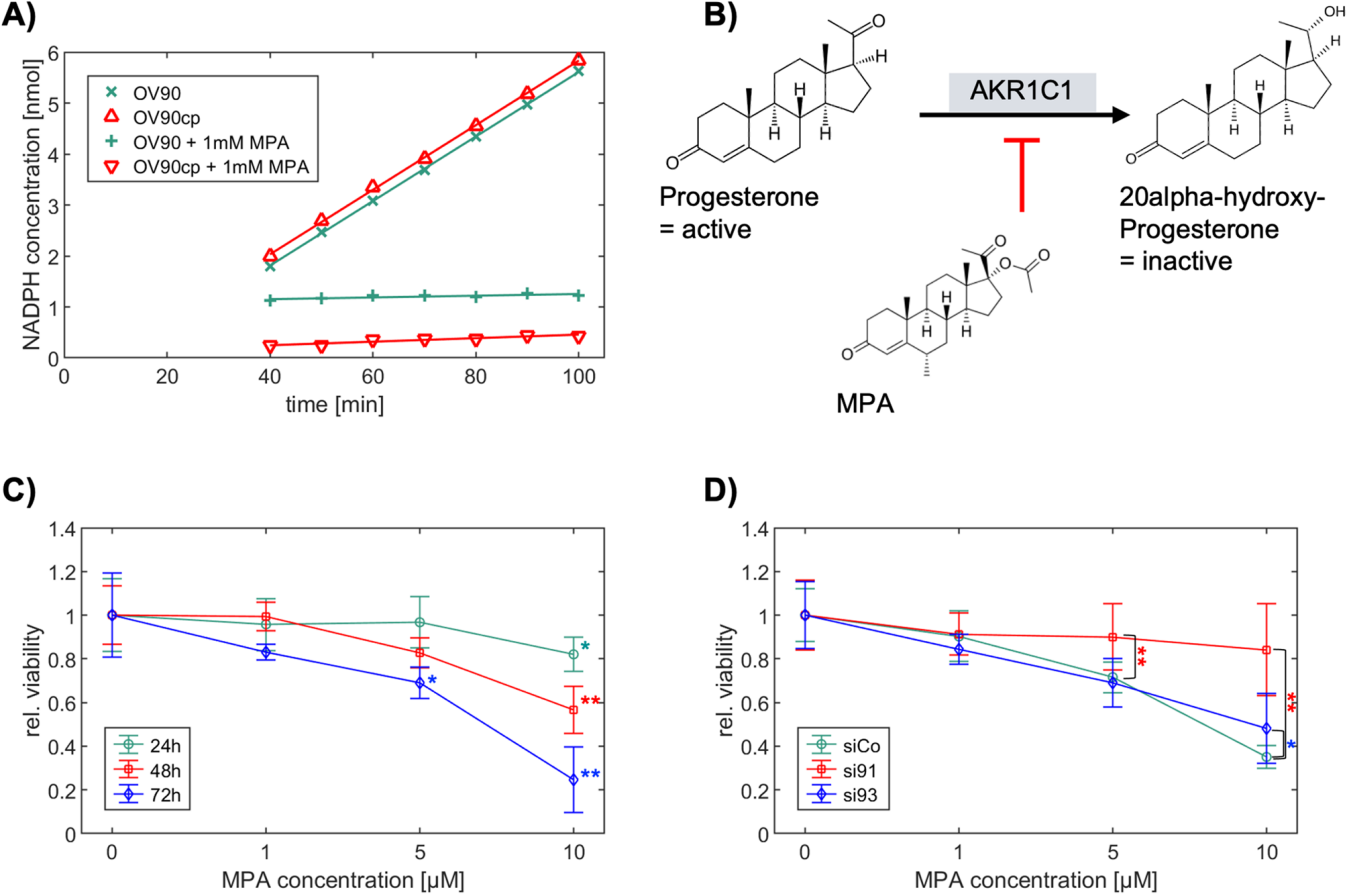
Effects of MPA treatment on AKR activity and OV90 viability. (**A**) NADPH formation as proportional measure to AKR activity, was determined colorimetrically in OV90/OV90cp cell lysates (10^6^ cells) in a time dependent manner. MPA administration leads to a potent inhibition of AKR activity (OV90: 97% inhibition; OV90cp: 94% inhibition). (**B**) AKR1C1 catalyzes preferentially the inactivation of progesterone^21^. The reaction is inhibited by MPA. (**C**) Viability of OV90 cells was reduced by MPA treatment in a concentration- and time-dependent manner (measured by MTT). Viability without treatment was used as comparison group for the statistical test. (**D**) *NFE2L2* silencing (24 h) weakens the effect of MPA treatment (48 h) on OV90 viability. Viability after *NFE2L2* knockdown was compared to the control transfected with scrambled siRNA at each MPA concentration for statistical analysis. *p < 0.05; **p < 0.01.

The progestin MPA used in the clinic is considered as pan-AKR1C inhibitor^21^. In our present study, kinetic measurements confirmed a potent inhibition of the AKR catalytic activity by MPA (> 90%; Fig. 4A). Furthermore, MPA treatment reduced OV90 viability in a concentration- and time-dependent manner (Fig. 4C). We then tested whether MPA acts via the NRF2-AKR1C1/2 signaling pathway. To do so, we silenced *NFE2L2* for 24 h with two specific siRNAs (si91 and si93, a scrambled siRNA served as control (siCo), Supplementary Fig. S5) and assessed the cell viability after 48 h treatment with MPA. We confirmed that the effects of MPA treatment were reduced after *NFE2L2* silencing (Fig. 4D), which indicates that NRF2-AKR1C1/2 are key factors for the molecular action of MPA.

### MPA sensitizes OV90/OV90cp towards carboplatin

**Figure 5:**
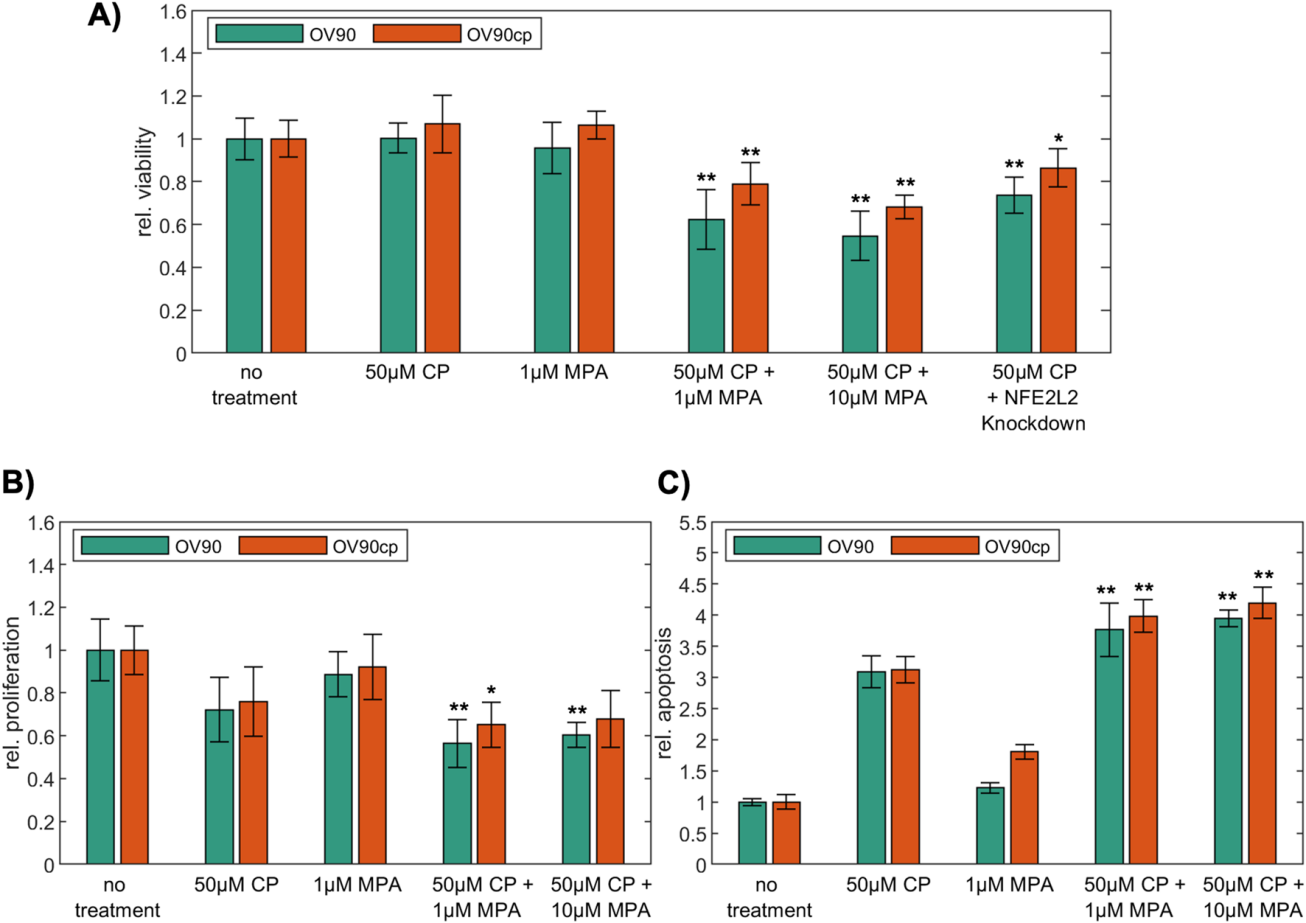
MPA sensitizes OV90/OV90cp towards carboplatin. (**A**) Viability (measured by MTT after 24 h treatment) and (**B**) proliferation (determined by BrdU assay after 48 h treatment) are significantly reduced by the combination of CP (50 µM) and MPA (1 and 10 µM) compared to single treatment. (**C**) A significantly increased apoptosis rate (measured by Cell Death Detection ELISA^PLUS^ 72 h after treatment) was observed for the combination therapy. T-test was applied to compare the combination therapy or *NFE2L2* silencing to CP monotherapy. *p < 0.05; **p < 0.01 (compared to 50 µM CP).

Since progesterone is reported to facilitate the toxicity of cisplatin in ovarian cancer cells^30^, we conducted functional assays to assess the therapeutic effect of CP and MPA as monotherapy and in combination on viability, proliferation, and apoptosis of OV90 and OV90cp cells. While 50 µM CP and 1 µM MPA as single agents showed no effect on viability after 24 h treatment, the combination of both therapeutics significantly reduced OV90/OV90cp viability by 38%/21% (Fig. 5A). Increased MPA concentration (10 µM) in combination with 50 µM CP further enhanced this effect (viability reduction of OV90/OV90cp by 45%/32%). *NFE2L2* silencing for 24 h induced a similar effect on OV90/OV90cp viability as AKR1C1/2 inhibition by MPA. These results confirm our hypothesis that active NRF2-AKR1C1/2 signaling is important for chemoresistance of OV90/OV90cp. We than tested the effect of CP and MPA on proliferation of the tumor cells. While proliferation of OV90/OV90cp was reduced to 72%/76% after 48 h CP monotherapy (Fig. 5B), the combination of CP with 1 µM MPA demonstrated a more substantial decrease in proliferation of cancer cells to 56%/65%. On proliferation the combination therapy with 1 µM MPA was as effective as with 10 µM MPA. To determine the effect of the combination therapy on apoptosis histone-associated DNA-fragments were quantified using a cell death detection ELISA. While CP monotherapy tripled the apoptosis rate after incubation of 72 h, the combination therapy led to a fourfold apoptosis rate (Fig. 5C). Taken together, our functional investigations show that AKR1C1/2 inhibition by MPA leads to increased response to CP in vitro.

## Discussion

In this translational study, AKR1C1/2 expression was firstly investigated by IHC in a well-established EOC collective. 82.1% of EOC specimens were positive for AKR1C1/2. Serous and endometrioid carcinomas showed significantly higher expression levels compared to other histological subtypes (Fig. 1). Strong positive correlations with the transcription factor NRF2 and the hormone receptors, including ERalpha, PGRA, and PGRB, suggest an interaction of NRF2-mediated signaling and hormone receptor-mediated signaling via AKR1C1/2 (Table 2). Consistent with our hypothesis that AKR1C1/2 is a key player in the development of chemoresistance, a significantly shorter PFS was noted for patients expressing AKR1C1/2 in their tumor tissue (Fig. 2A). In the second part, in vitro experiments revealed an induction of AKR1C1/2 in a platinum resistant cell culture model (Fig. 3B). Treatment with MPA led to a potent inhibition of AKR enzyme activity and to a time- and concentration-dependent decline of OV90 viability, which was attenuated by *NFE2L2* silencing (Fig. 4). Combination therapy with CP and MPA reduced viability and proliferation and enhanced apoptosis of OV90/OV90cp significantly compared to CP monotherapy (Fig. 5). Increased MPA concentration in combination with CP further reduced OV90/OV90cp viability. A comparable effect on OV90/OV90cp viability was elicited by *NFE2L2* silencing, reinforcing the importance of the NRF2-AKR1C1/2 pathway in chemoresistance.

**Figure 6:**
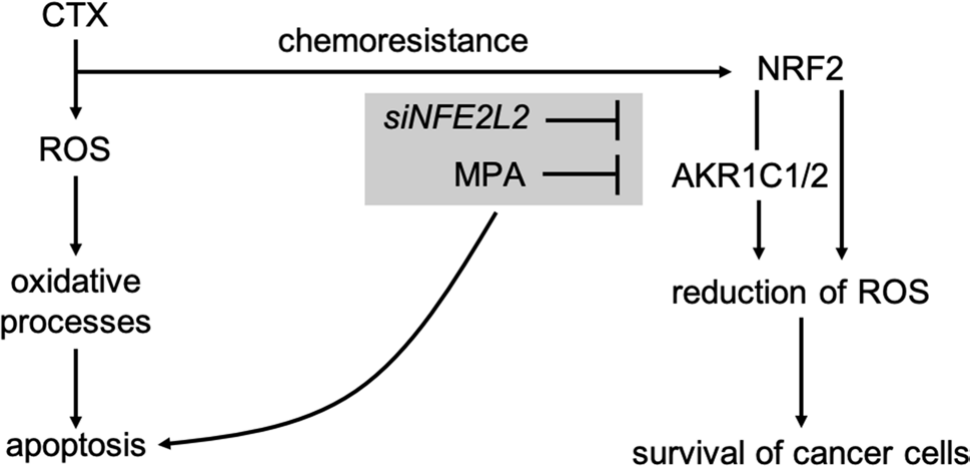
Role of AKR1C1/2 in chemoresistance. Chemotherapeutics (CTX) generate oxidative stress, which contributes significantly to the destruction of tumor cells (left pathway). In case of chemoresistance, NRF2-mediated antioxidant response pathway mitigates the effect of CTX via AKR1C1/2 and other target genes. ROS are reduced by AKR1C1/2, which promotes the survival of cancer cells (right pathway). Inhibition of this pathway either by *NFE2L2* silencing or AKR1C1/2 inhibition by MPA (grey box) resensitizes ovarian cancer cells to CTX and results in an increased apoptosis rate.

As schematic representation of the study Fig. 6 shows the role of AKR1C1/2 in context of chemoresistance. Oxidative stress, which is generated by chemotherapeutic agents and which consequently leads to cell death, is reduced by the NRF2-mediated pathway via its effector proteins^6,7^. In particular, AKR1C1/2 detoxify ROS which leads to cancer progression^18^. We show that an inhibition of this pathway either by *NFE2L2* silencing or direct AKR1C1/2 inhibition by MPA resensitizes ovarian cancer cells to CTX and results in reduced viability, proliferation, and an increased apoptosis rate.

In healthy tissue NRF2 acts as tumor suppressor, protecting cells from xenobiotic and oxidative stress. Conversely, several studies report hyperactivation of NRF2 signaling in cancer and its negative prognostic effect^8–16,42^. In addition, an overexpression of its target genes *AKR1C1/2* has been described in different cancer types^19,22,43^. A previously published study investigating AKR1C in EOC by IHC revealed shorter PFS for patients with AKR1C expression, which did not reach statistical significance due to a small collective (44 patients)^44^. In our collective, we verified the negative prognostic effect of AKR1C1/2 expression on PFS. This may be caused by the development of chemoresistance. Emerging evidence points to a relevant contribution of NRF2-AKR1C1/2 in this process. In endometrial cancer, NRF2-AKR1C1 overexpression was proposed to be the underlying mechanism of progestin resistance^23^. AKR1C expression in T-ALL cells of therapy resistant patients correlates with response to chemotherapeutics^45^. Accordingly, a cDNA microarray analysis identified *AKR1C* as one of five differentially expressed genes in the platinum resistant variant of the ovarian cancer cell line 2008^46^. Ovarian cancer cells (2008, SKOV3 and A2780) show an increased platinum resistance by *AKR1C1/2* overexpression^46,47^. Chen et al. demonstrated that an overexpression of *AKR1C1-3* decreases ROS levels, while *AKR1C1/2* knockdown reduces platinum resistance in 2008 cells^48^. Consistently, in our study carboplatin treatment resulted in an induction of AKR1C1/2 expression in A2780cp and OV90cp cells. A comparison of the IC50 values confirmed the chemoresistance of these clones. OV90 and SKOV3 cells have been described as resistant ovarian cancer cell lines before showing higher levels of NRF2 and target genes than A2780^40^. This report corresponds to our in vitro results and was extended by AKR activity measurements, which show higher AKR enzyme activity of OV90 compared to A2780. However, a clear upregulation of NRF2 in the resistant clones could not be shown. A reason could be either that transcription factors like NRF2 have shorter half-life than the effector proteins like AKR1C1/2 or AKR1C1/2 may be influenced by other regulatory mechanisms. By *NFE2L2* silencing, Manandhar et al. describe a sensitization of OV90 cells towards doxorubicin^49^, while we show a sensitization towards CP. Thus, NRF2-AKR1C1/2 enhances resistance to several chemotherapeutic agents.

In addition to conventional CTX, endocrine therapy based on hormone receptor expression should be reconsidered for EOC treatment. Both, in vitro and clinical data suggest a protective role of progesterone and progestins in EOC^50^. Progesterone receptor expression in EOC was associated with improved OS and PFS^51^, which may be mediated by antiproliferative and pro-apoptotic properties of progesterone^50^. In our study, the progestin MPA led to a time- and concentration dependent decline of OV90 viability. Inhibitory effects of high dose MPA (10 µM) have been noted before in ovarian cancer cells (SKOV3 and OVCAR3)^52,53^ and could be either caused by direct cytotoxicity or decreased proliferation via the progesterone receptor and lower gonadotropin and estrogen levels^54^. Importantly, there is some evidence that MPA reverses chemoresistance^30,45,55^. The increased response of OV90/OV90cp to CP in combination with MPA, which has been shown in this study, can be attributed to inhibitory effects of MPA on AKR enzyme activity.

Our in vitro data suggest that the combination of MPA and CP might be a promising therapeutic approach to increase response rates of EOC patients. As endocrine therapy, MPA is not only less toxic than chemotherapeutic agents but also mediates myeloprotective effects and corticoid-like effects leading to weight gain^54^. The therapeutic use of MPA in EOC has already been investigated in small clinical trials^56,57^. While high dose MPA treatment of patients with resistance to CTX led only to partial response in 15% of investigated cases^56^, a pilot study investigating the effects of low dose MPA plus platinum-based chemotherapy on advanced EOC (FIGO Stage III/IV) reports significantly improved survival as well as recurrence rates^57^.

## Conclusions

Our data confirm that AKR1C1/2 is a key player in the development of chemoresistance and an independent indicator for short PFS. Inhibition of AKR1C1/2 by MPA leads to an increased response to CP in vitro. Thus, a combination therapy with CP and MPA might be a therapeutic approach to increase response rates of EOC patients. Since hormone therapy is well tolerated and a pilot study already showed promising results in advanced EOC, the combination of CP and MPA should be explored in a wider clinical context e.g., a randomized controlled study.

## Supplementary Information

Supplementary Information.

## Abbreviations

ACTB: Actin beta
AKR1C1/2: Aldo–keto reductase family 1 member C1 and 2
BrdU: 5-Bromo-2′-deoxyuridine
CP: Carboplatin
CTX: Chemotherapeutics
EOC: Epithelial ovarian cancer
FIGO: Federation of Gynecology and Obstetrics
GAPDH: Glyceraldehyde 3-phosphate dehydrogenase
IC50: Half maximal inhibitory concentration
IHC: Immunohistochemistry
IQR: Interquartile range
IRS: Immunoreactive score
MPA: Medroxyprogesterone acetate
MTT: 3–(4,5-Dimethylthiazol-2-yl)-2,5-diphenyltetrazolium bromide
NADPH: Nicotinamid-adenin-dinucleotid-phosphat
NFE2L2/NRF2: Nuclear factor E2-related factor 2
norm. optical density: Normalized optical density
OD: Optical density
OS: Overall survival
PFS: Progression free survival
rel. apoptosis: Relative apoptosis
rel. expression: Relative expression
rel. proliferation: Relative proliferation
rel. viability: Relative viability
ROC: Receiver operating characteristic
ROS: Reactive oxygen species
siRNA: Small interfering ribonucleic acid
TNM: Tumor (T), nodes (N), and metastases (M) classification of malignant tumors
