# Supplementary material for: AKR1C1/2 inhibition by MPA sensitizes platinum resistant ovarian cancer towards carboplatin
Source: Sci Rep. 2022 Feb 3;12:1862. doi: 10.1038/s41598-022-05785-9 (PMC8814148; doi:10.1038/s41598-022-05785-9)
Supplement: Supplementary file 1 — Supplementary Information. [file 41598_2022_5785_MOESM1_ESM.pdf]

Supplementary Information

AKR1C1/2 inhibition by MPA sensitizes platinum resistant ovarian cancer towards carboplatin

Susann Badmann[1], Doris Mayr[2], Elisa Schmoeckel[2], Anna Hester[1], Christina Buschmann[1], Susanne Beyer[1], Thomas Kolben[1], Fabian Kraus[1], Anca Chelariu-Raicu[1], Alexander Burges[1], Sven Mahner[1], Udo Jeschke[1,3], Fabian Trillsch[1], *Bastian Czogalla[1]

[1]Department of Obstetrics and Gynecology, University Hospital, LMU Munich, Munich, Germany;

[2]Institute of Pathology, Faculty of Medicine, LMU Munich, Munich Germany;

[3]Department of Obstetrics and Gynecology, University Hospital Augsburg, Augsburg, Germany;

*Corresponding author: bastian.czogalla@med.uni-muenchen.de

Supplementary Figures

A) B)

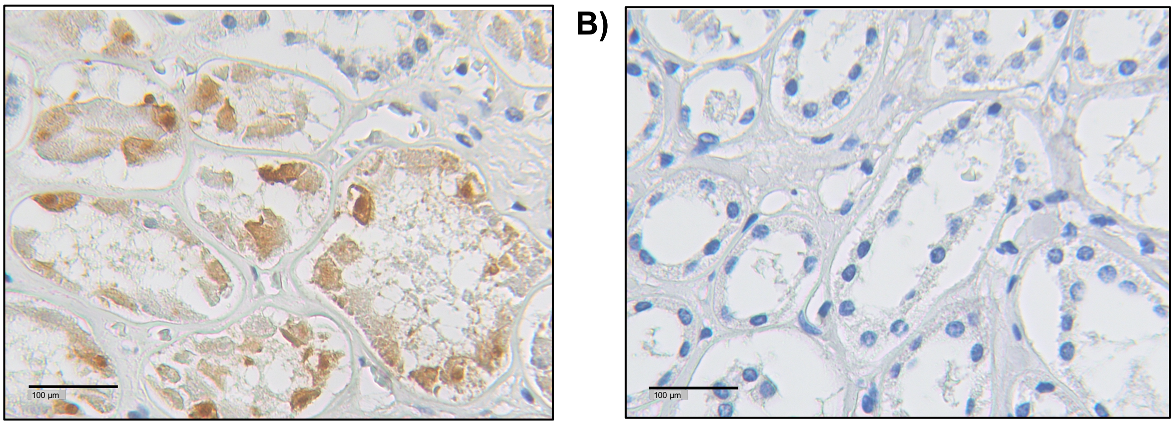

Figure S1. Positive (A) and negative (B) system control for AKR1C1/2 staining in kidney (25x magnification, scale bar=100 µm).

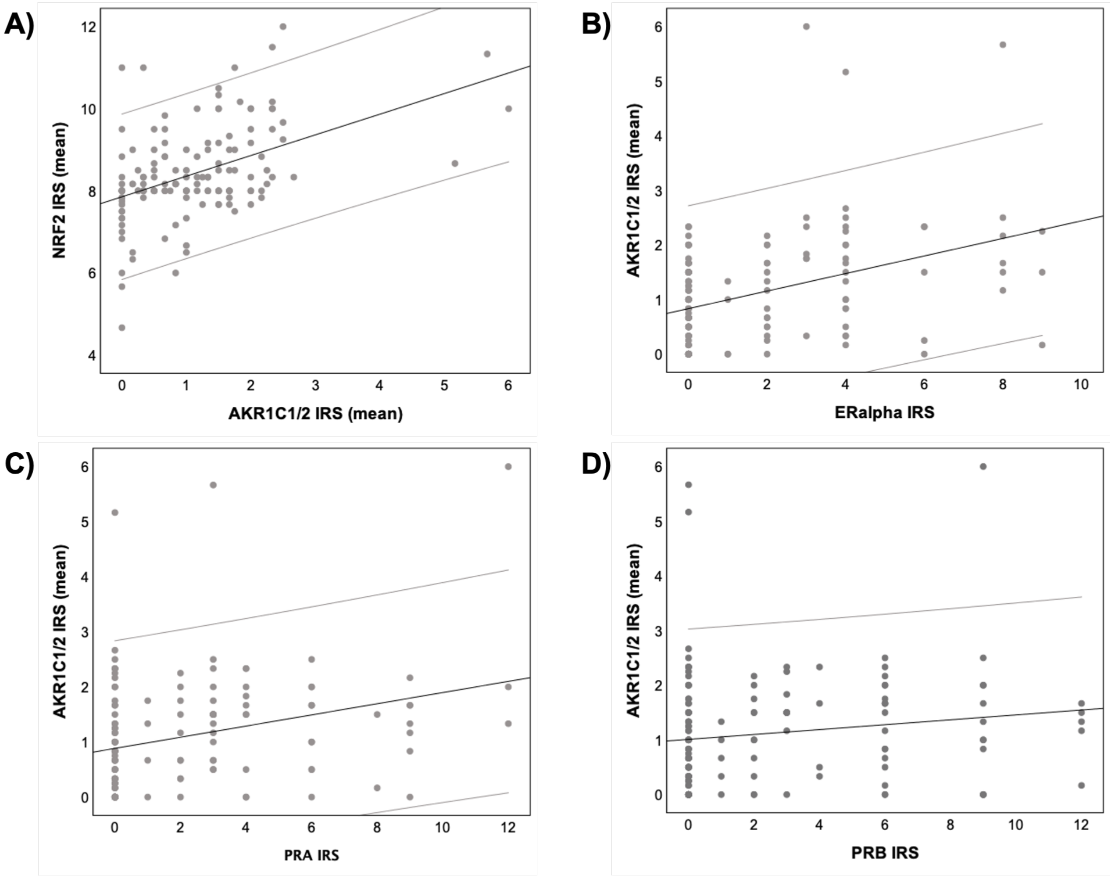

Figure S2. Graphic representations of the correlations between NRF2, AKR1C1/2 and the hormone receptors shown in Table 2 of the main manuscript. IRS of NRF2 (mean) and AKR1C1/2 (mean) were correlated to each other and to the IRS of hormone receptors using Spearman`s correlation analysis. A linear fit as well as a 95% confidence interval were inserted into the scatter plots.

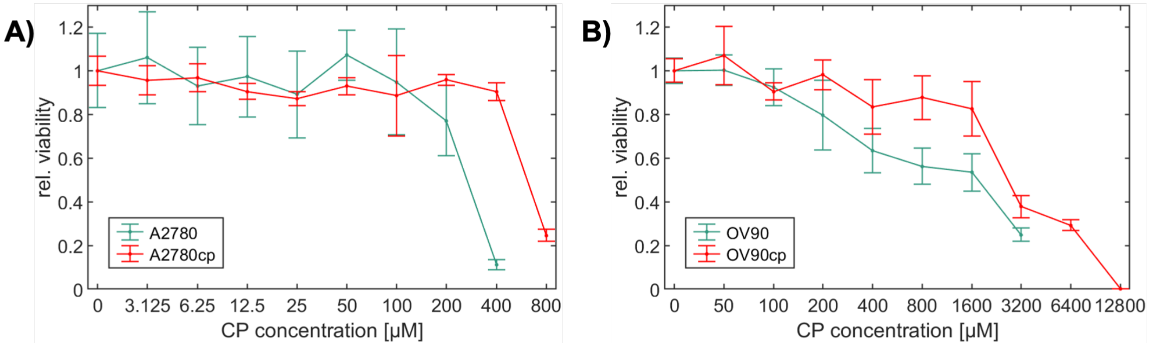

Figure S3. OV90/OV90cp (B) is more resistant towards CP than A2780/A2780cp (A). The viability was measured by MTT assay after 24h treatment with raising CP concentrations. To assess the chemoresistance of the cell lines, the IC50 value was determined (see Table 4).

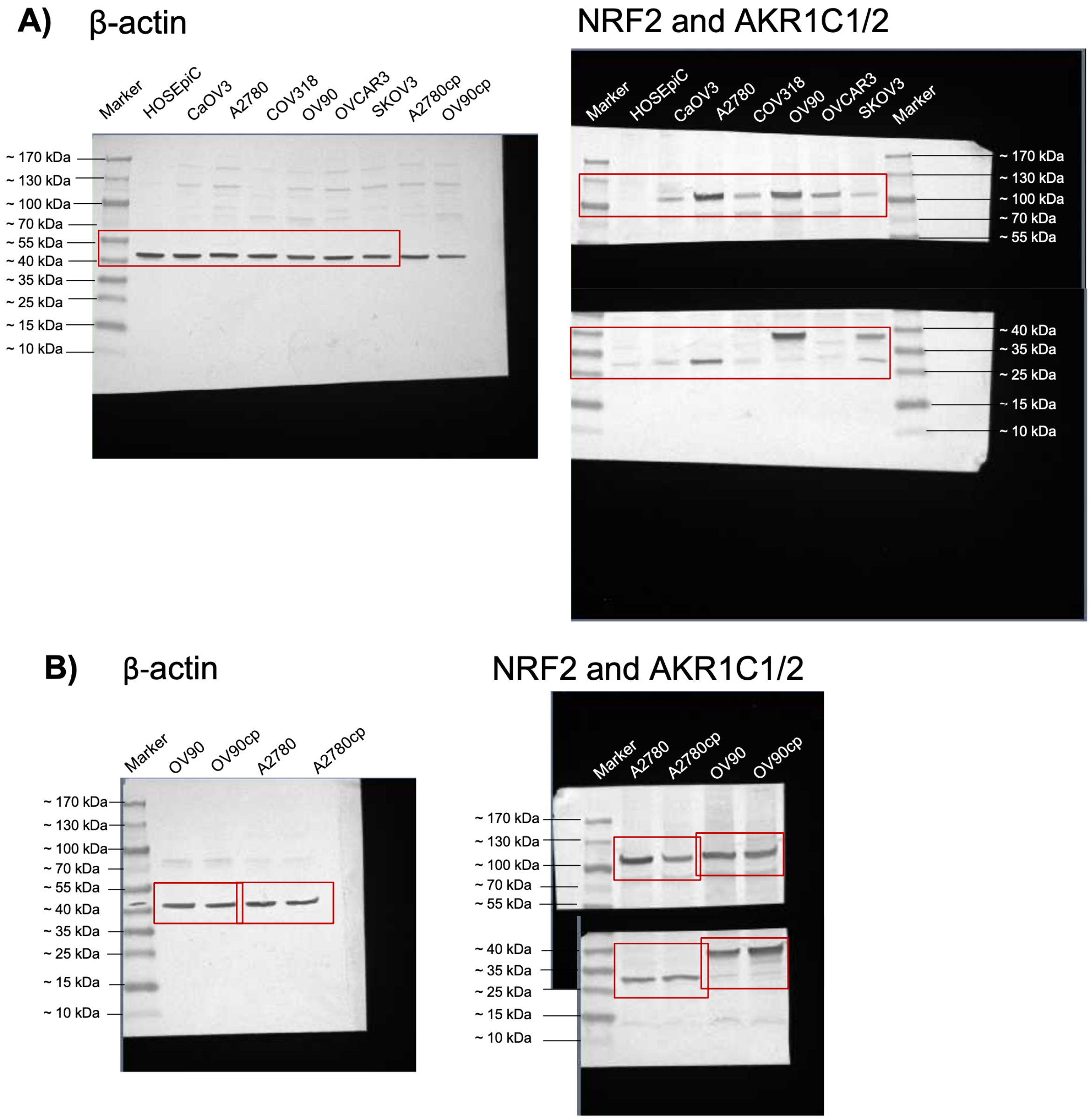

Figure S4. Full-length western blots of Figure 3 main manuscript (red boxes). A) Basal expression of NRF2 and AKR1C1/2 of ovarian cancer cell lines compared to the benign ovarian epithelial cell line HOSEpiC. B) Protein expression of NRF2 and AKR1C1/2 of the endometrioid EOC cell line A2780 and the serous EOC cell line OV90 and their resistant clones. β-actin served as control. Western blot detection was performed with VECTASTAIN ABC-AmP Reagent (Vector Laboratories, Burlingame, CA, USA). Pictures were captured with Bio-Rad Universal Hood II (Bio-Rad Laboratories Inc., Hercules, CA, USA) and the corresponding Software Quantity One enabled a quantitative analysis of the blots.

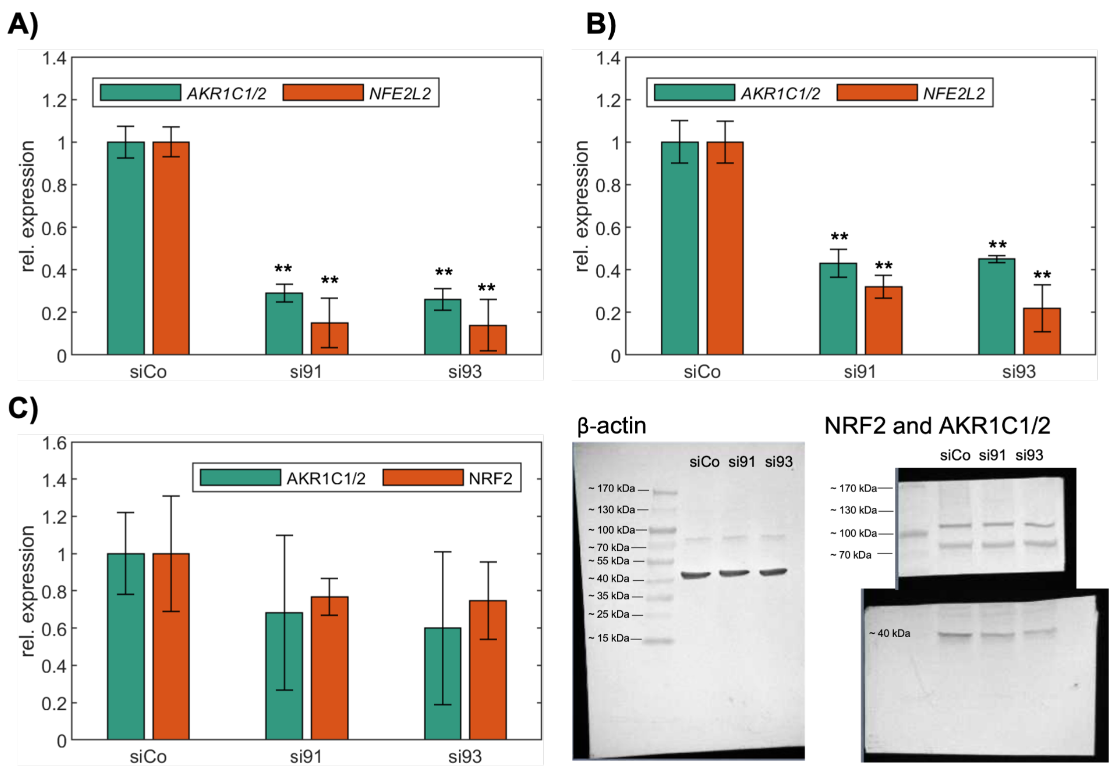

Figure S5. Successful downregulation of NRF2 and AKR1C1/2 by siRNA knockdown. The efficiency of *NFE2L2* silencing was investigated on mRNA level by qPCR in OV90 (A) and OV90cp cells (B) and on protein level by western blot analysis (OV90, C) 24h after transfection of si*NFE2L2* (**: p<0.01).

Supplementary Tables

Table S1. Descriptive statistics of expression analysis.

**AKR1C1/2**		**NRF2**	
**Total**	140	**Total**	150
**IRS>0**	115	**IRS>0**	150
**Mean IRS**	1.12	**Mean IRS**	8.36
**Median IRS**	1	**Median IRS**	8
**IRS Range**	0-6	**IRS Range**	4-12
**IRS**	**Frequency**	**IRS**	**Frequency**
0	25	4	1
0.17	5	4.67	1
0.25	3	5.67	1
0.33	8	6	2
0.5	9	6.33	2

0.67	9	6.5	3
0.75	1	6.67	2
0.83	6	6.83	2
1	10	7	2
1.17	5	7.17	2
1.25	2	7.33	3
1.33	6	7.5	3
1.5	12	7.67	6
1.67	8	7.75	1
1.75	4	7.83	6
1.83	1	8	33
2	8	8.17	10
2.17	3	8.33	12
2.25	2	8.5	10
2.33	6	8.67	3
2.5	3	8.83	5
2.67	1	9	6
5.17	1	9.17	4
5.67	1	9.25	1
6	1	9.33	1
		9.5	6
		9.67	1
		9.83	1
		10	9
		10.17	2
		10.33	1
		10.5	2
		11	3
		11.33	1
		11.5	1
		12	1

Table S2. Antibodies used for western blotting.

Antibody	Dilution	Manufacturer
**NRF2, monoclonal rabbit**	1:50	abcam, Cambridge, UK
**AKR1C1/2, polyclonal rabbit**	1:50	Sigma-Aldrich Co., St. Louis, MO, USA
**β-Actin, monoclonal mouse**	1:1000	Sigma-Aldrich Co., St. Louis, MO, USA

Table S3. Sequences of primers used in qPCR to determine mRNA expression levels.

NFE2L2	forward: CAC GGT CCA CAG CTC ATC ATG
	reverse: GAT CTA TAT CTT GCC TCC AAA GTA TGT C
AKR1C1/2	forward: CAGCAGTGCGAGGGTCAGAG
	reverse: GCCTTCCCATACCTGACTTCTAATC
ACTB	forward: TCCTCCCTGGAGAAGAGCTA
	reverse: CGTGGATGCCACAGGACT
GAPDH	forward: AGCCACATCGCTCAGACAC
	reverse: GCCCAATACGACCAAATCC
